# Small RNA-seq reveals novel regulatory components for apomixis in *Paspalum notatum*

**DOI:** 10.1186/s12864-019-5881-0

**Published:** 2019-06-13

**Authors:** Juan Pablo A. Ortiz, Olivier Leblanc, Cristian Rohr, Mauricio Grisolia, Lorena A. Siena, Maricel Podio, Carolina Colono, Celeste Azzaro, Silvina C. Pessino

**Affiliations:** 1 Instituto de Investigaciones en Ciencias Agrarias de Rosario (IICAR, CONICET-UNR), Facultad de Ciencias Agrarias, Universidad Nacional de Rosario, Zavalla, Argentina; 2 Institut de Recherche pour le Développement (IRD), Université de Montpellier, Montpellier, France; 3 Instituto de Agrobiotecnología de Rosario (INDEAR), Rosario, Argentina

**Keywords:** Apomixis, Apospory, Auxin, miRNA, Plant reproduction, sRNA

## Abstract

**Background:**

Apomixis is considered an evolutionary deviation of the sexual reproductive pathway leading to the generation of clonal maternal progenies by seeds. Recent evidence from model and non-model species suggested that this trait could be modulated by epigenetic mechanisms involving small RNAs (sRNAs). Here we profiled floral sRNAs originated from apomictic and sexual *Paspalum notatum* genotypes in order to identify molecular pathways under epigenetic control that might be involved in the transition from sexuality to agamospermy.

**Results:**

The mining of genes participating in sRNA-directed pathways from floral *Paspalum* transcriptomic resources showed these routes are functional during reproductive development, with several members differentially expressed in apomictic and sexual plants. Triplicate floral sRNA libraries derived from apomictic and a sexual genotypes were characterized by using high-throughput sequencing technology. EdgeR was apply to compare the number of sRNA reads between sexual and apomictic libraries that map over all *Paspalum* floral transcripts. A total of 1525 transcripts showed differential sRNA representation, including genes related to meiosis, plant hormone signaling, biomolecules transport, transcription control and cell cycle. Survey for miRNA precursors on transcriptome and genome references allowed the discovery of 124 entities, including 40 conserved and 8 novel ones. Fifty-six clusters were differentially represented in apomictic and sexual plants. All differentially expressed miRNAs were up-regulated in apomictic libraries but miR2275, which showed different family members with opposed representation. Examination of predicted miRNAs targets detected 374 potential candidates. Considering sRNA, miRNAs and target surveys together, 14 genes previously described as related with auxin metabolism, transport and signaling were detected, including *AMINO ACID/AUXIN PERMEASE 15*, *IAA-AMIDO SYNTHETASE GH3–8*, *IAA30*, miR160, miR167, miR164, miR319, *ARF2*, *ARF8*, *ARF10*, *ARF12*, *AFB2*, *PROLIFERATING CELL FACTOR 6* and *NITRATE TRANSPORTER 1.1*.

**Conclusions:**

This work provides a comprehensive survey of the sRNA differential representation in flowers of sexual and apomictic *Paspalum notatum* plants. An integration of the small RNA profiling data presented here and previous transcriptomic information suggests that sRNA-mediated regulation of auxin pathways is pivotal in promoting apomixis. These results will underlie future functional characterization of the molecular components mediating the switch from sexuality to apomixis.

**Electronic supplementary material:**

The online version of this article (10.1186/s12864-019-5881-0) contains supplementary material, which is available to authorized users.

## Background

In plants, several small RNA pathways that likely diversified from a cellular defense mechanism against viruses and transposable elements are essential regulators of genome expression through mRNA cleavage, translational repression and DNA methylation [1]. As such, small RNAs perform critical functions in development, stress responses, and transgenerational inheritance [2]. In particular, they contribute to numerous aspects of sexual reproduction [3, 4]. Moreover, several alterations in either small RNA biogenesis, processing or targeting partially phenocopy apomixis [5], i.e. a group of peculiar reproductive behaviors leading to the formation of maternal progeny within seeds [6].

Currently, apomictic developments are viewed as the outcome of the deregulation of one or more developmental program/s involved in the sexual pathway [7], and they are broadly categorized into adventitious embryony and gametophytic apomixis [8]. Adventitious embryony relates to the spontaneous generation of somatic embryos from the maternal tissues involved in seed formation, whereas gametophytic apomixis encompasses the formation of functional, unreduced (2n) embryo sacs (2n-ES) from either diploid megaspore mother cells (diplospory) or nucellar companion cells (apospory) [8]. Within 2n-ES, unreduced egg cells develop parthenogenetically into maternal embryos, while the formation of viable seeds ultimately depends on endosperm development after central cell fertilization (pseudogamy) or, more sporadically, autonomous divisions [8]. Therefore, since seeds are formed in absence of meiosis and egg cell fertilization, they contain clonal embryos genetically identical to the mother plant.

Considering the prospects for the permanent fixation of any genotype regardless of genetic complexity, harnessing apomixis into agriculture would allow to dramatically accelerate plant breeding schemes and reduce the cost of improved varieties [9]. However, all major food crops reproduce sexually and attempts to introduce the trait from wild relatives in maize and pearl millet have remained unsuccessful, highlighting our lack of knowledge for apomixis molecular determinants [10–12]. Such outcome strongly contrasts with the common view of independent and repeated emergence of the trait during angiosperm evolution, therefore suggesting relatively simple controlling mechanisms [13]. A likely explanation for this is the complex nature of both apomictic plant genomes (i.e. highly heterozygous and polyploid) and apomixis-linked regions (i.e. hemizygosity, restricted recombination and heterochromatinization) ([14], and references therein). Therefore, attempts to identify candidate genes through map-based cloning approaches have proven difficult [15], however they succeeded in isolating key genes such as *BABY BOOM-like* controlling parthenogenesis in aposporous *Pennisetum squamulatum* [16], *ORIGIN RECOGNITION COMPLEX3* promoting imbalanced endosperm development in aposporous *Paspalum simplex* [17] or *QUI GON JINN* necessary for the emergence of 2n-ES in aposporous *Paspalum notatum* [18]. On the other hand, transcriptome comparisons between apomictic and sexual individuals have already uncovered many differentially expressed genes and several molecular pathways related to apomixis including, among others, protein degradation, transcriptional regulation, stress response, and cell-to-cell signaling [19–24].

Although a large amount of information indicates that apomixis is genetically determined [12], increasing evidence from model and apomictic species suggests that the disruption of epigenetic mechanisms participating in the control of sexual and asexual reproduction may mediate the switch between both types of reproduction [5, 25, 26]. First, 5-azacytidine induced DNA demethylation produced a significant reduction in parthenogenesis in aposporous *P. simplex* [27]. Moreover, increased sexuality was detected in diplosporous *Eragrostis curvula* plants subjected to environmental stress [28, 29]. Furthermore, among candidate mechanisms mediating apomixis, dosage effects or short-term epigenetic responses following polyploidization [30–32], and trans-effects generated by a non-recombinant, specific region in apomict genomes [17] have been proposed. In addition, genetic analyses in *Arabidopsis thaliana* and maize have shown that inactivation of members of the RNA-dependent DNA Methylation (RdDM) pathway results in reproductive phenotypes reminiscent of apomictic developments [33–36]. Interestingly, in diplosporous *Boechera holboelli* and *E. curvula* RdDM members are deregulated in ovules of apomictic plants compared to sexual plants [33, 37].

These observations led several research groups to explore small RNA components in reproductive organs of sexual and apomictic plants belonging to different genera, including diplosporous *Boechera* [38], aposporous *Hypericum* and *Hieracium* [39, 40], and *Citrus* (which shows adventitious embryony) [41]. These works identified the main conserved plant miRNA families and their target genes, as well as a novel family in *Citrus* (miRN23-5p) [38–41]. Although deregulation of some precursors was reported, the role for miRNAs in apomixis remains elusive, since only a few targets showed a congruent expression pattern, such as the upregulation of *SQUAMOSA PROMOTER BINDING PROTEIN LIKE11* and *Cs9g06920* (an unknown protein containing the XS conserved domain) after miR156/157 (*Boechera*) and miRN23-5p (*Citrus*) decreased expression in apomictic ovules. Similarly, transcript expression differences between sexual and apomictic *Hieracium* ovules correlated poorly with 24-nt small RNAs abundances, since only two *EXORDIUM-like* genes could be identified among 40 differentially expressed targets [40].

The *Paspalum* genus is an attractive biological system for studying apomixis, since it is both a model system for mining candidate gene(s) and an important target forage crop [42]. The sophisticated reproductive strategy used by *Paspalum* spp. (*i. e.* a combination of sexuality, apomixis and vegetative propagation), together with the existence of a wide range of cytotypes (with ploidy levels ranging from 2x to 16x), likely contributed to complex functional diversification and wide distribution across the Americas, from North Patagonia to Central Mexico [42]. Moreover, although *Paspalum* species are a key endemic forage resource for South American grasslands and pasture agro-ecosystems, they remain largely undomesticated. Therefore, generating novel knowledge on the genetic and molecular determinants of apomixis is essential for breeding and delivering novel *Paspalum* cultivars adapted to the challenges of climate change and societal demands on agriculture [24, 42]. To achieve this, we completed the existing transcriptomic resources available for *P. notatum* [24] with small RNA-seq profiling in florets of sexual and apomictic plants. Both transcriptomic data and genome information available in Gramineae species for miRNA gene annotation and quantitation were used to establish a comparative approach. We present evidence for differences in small RNAs profiles, including the identification of novel miRNA families, which might contribute to explain the differentiation between sexuality and apomixis.

## Results and discussion

### Identification of *Paspalum* genes involved in key small RNA pathways

Small-RNA directed pathways all rely on the activities of DICER-LIKE (DCL) enzymes that generate 21–24-nt small RNAs by processing: 1) hairpins of single-stranded RNAs synthesized from RNA POLYMERASE II (Pol II); 2) long double-stranded RNAs (dsRNAs), whose biogenesis requires transcription by Pol II or RNA POLYMERASE IV (Pol IV) followed by dsRNA synthesis by RNA-DEPENDENT RNA POLYMERASEs (RDRs); and 3) long dsRNAs arising from the hybridization of sense and antisense transcripts, from the fold-back of an inverted-repeat sequences, or from the hybridization of unrelated RNA molecules with sequence complementarity [1]. Following biogenesis, small RNAs are loaded onto ARGONAUTE proteins (AGO), which mediate targeting of complementary RNAs or DNAs, resulting in post-transcriptional gene silencing (PTGS; degradation through cleavage or translational repression) or RNA-dependent DNA methylation (RdDM) through the recruitment of DNA methylation factors [1, 2]. Therefore, AGO proteins are central in plant small RNA pathways, owing to extensive diversification during evolution and providing specialization to the various classes of small RNAs [1, 43].

**Fig. 1:**
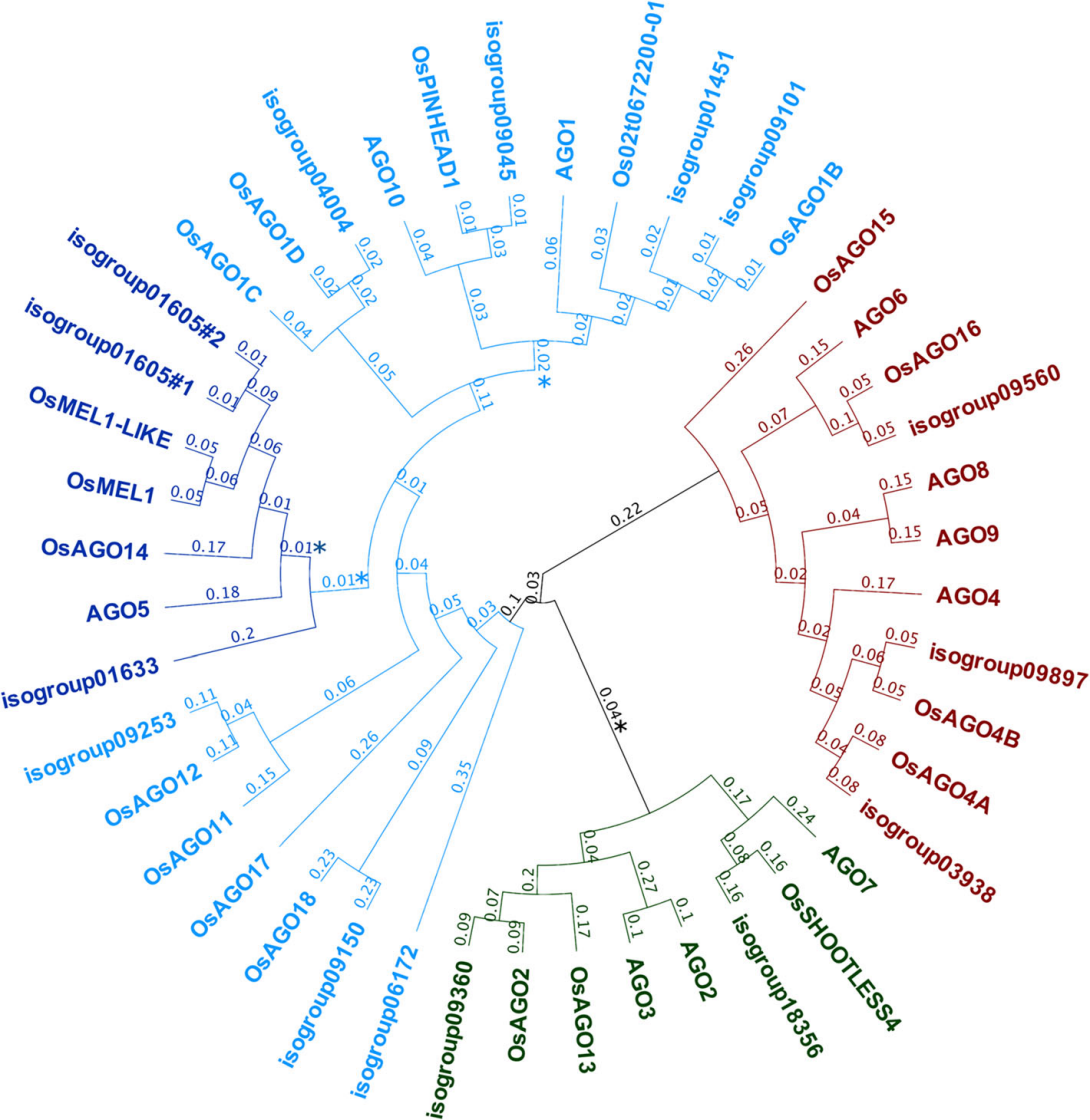
Phylogenetic analysis of AGO family proteins of *A. thaliana*, *O. sativa* Japonica, and *P. notatum. A. thaliana* and *O. sativa* sequences were obtained from the Gramene database [76]. *P. notatum* sequences were retrieved from the corresponding floral reference transcriptome [24] Sequences alignment and neighbor-joining tree construction was performed by using Geneious 10.0.9. Branches with bootstrap support values < 50% are indicated by a star (1000 replicates). Substitution rates per site values are shown for each branch

In order to evaluate the role of sRNA-directed pathways in apomixis, we first mined previous *Paspalum* reference floral transcriptome annotations [24] for genes related to sRNA biogenesis and function. Surveys for AGO rice homologs revealed 22 transcripts, which assembled into 15 genes (Additional file 1, section 1). Transcripts assembled into isotigs usually showed polymorphisms (both INDELs and SNPs) and might be allelic variants of the same gene, however some variations suggest they derive from genes existing in multiple copies in the *P. notatum* genome (i.e. isotigs of isogroup 01605). Predicted *P. notatum* AGO proteins (PnAGOs) were aligned with *O. sativa* Japonica and *A. thaliana* AGO proteins and a phylogenetic tree was inferred after careful visual inspection and post-processing multiple alignments for masking gaps. As shown in Fig. 1, PnAGOs distributed into the three main classes of plant AGOs. Homologs of AGO involved in meiosis (MEL1) and in reproductive cell fate specification (AGO4, AGO9) were identified. Similarly, we next identified *P. notatum* transcript homologs to most of the genes encoding proteins involved in key phases of PTGS and RdDM [44] including: Pol IV-dependent small RNA biogenesis, Pol V-dependent de novo DNA methylation, chromatin alterations and DNA methylation (*n* = 67 hits) (for details see Additional file 1, section 1). Note that, despite using less stringent criteria for filtering Blastx results, we identified no homolog for RDR2, a key member of RNA biogenesis in the RdDM pathway. Furthermore, differential expression analysis of the detected orthologs between sexual and apomictic florets in the reference transcriptome showed significant variation for four transcripts out of all identified, including: decreased expression for a transcript predicted to encode for OsAGO1D (isotig18136; FDR = 0.00091 and FC > 3.24) and increased expression for transcripts predicted to encode Os HISTONE DEACETYLASE 705 (isotig09390 and isotig09392; FDR < 1e-03; FC > 30) and OsDCL1 (isotig27521; FDR = 0.0048, FC > 1.8) in the apomictic library respect to the sexual one (Additional file 1, section 2). In conclusion, our analysis strongly suggests that small RNA-directed pathways are likely functional in *P. notatum* floral tissues and show differential modulation in apomictic and sexual pathways.

### Mapping of sRNA reads onto the *P. notatum* floral transcriptome

**Fig. 2:**
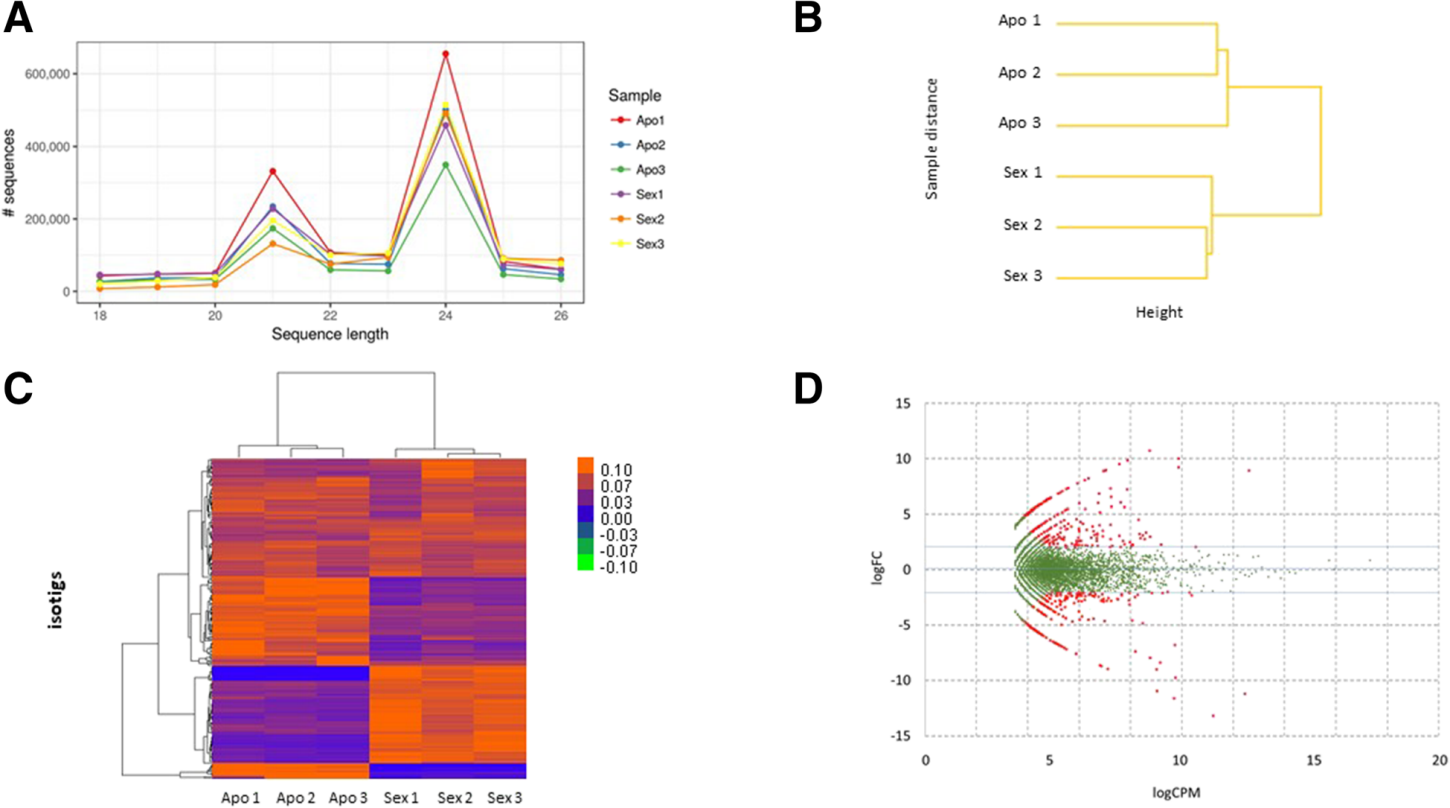
General analysis of sRNA sequencing outputs derived from sexual and apomictic *P. notatum* floral samples. **a** Length distribution of small RNAs sequences after trimming and adapter removal. **b** Clustering of the number of small RNA sequences aligned to a *Paspalum* floral transcriptome reference obtained from the three replicates of sexual and apomictic samples. **c** Heat map of the 250 transcripts carrying the largest number of small RNA reads. Orange and blue colours indicate higher and lower numbers of sRNAs mapping in each transcript, respectively. **d** Mean-difference plot for differential small RNA transcripts coverage in sexual and apomictic floral tissues. Only transcripts carrying *n* > 5.0 were considered. Green dots: non-significant differential expression. Red dots: significant differential expression (logFC >|2|, FDR < 0.05). Positive logFC: upregulation in apomictic samples. Negative logFC: upregulation in sexual samples

Next, we decided to characterize the floral sRNA transcriptome components of an apomictic (Q4117) and a sexual (C4-4x) genotype, in mixed samples representing four developmental stages, by using a triplicate Illumina small RNA sequencing assay. The total number of raw reads across libraries ranged from 1,558,547 to 2,996,675, with an average % GC of 54.67 ± 0.51 (Additional file 2). Raw read controls revealed satisfactory quality levels, since mean Phred quality scores were between 33.2 and 38.1 along all nucleotide (nt) positions, with most sequences displaying scores between 36 and 38. A considerable number of sequences were duplicated at levels ranging from 10 to 104 and the percentage of undetermined bases at each position was near 0 for all samples. Table 1 displays the number of filtered reads (18–26 nt) for each sample. All samples showed similar length distributions, with two peaks at 21 nt and 24 nt (Fig. 2a).

**Table 1:** General statistics of the *P. notatum* sRNA reads derived from apomictic and sexual libraries

Library	Total number of selected reads	Sequences flagged as poor quality	% Duplicated sequences (average)	Sequence length	% GC content (average)
Apo1_S1	1,479,243	0	65.4	18–26	57.0
Apo2_S2	1,096,989	0	60.9	18–26	56.0
Apo3_S3	810,471	0	59.2	18–26	56.0
Sex1_S4	1,162,189	0	64.5	18–26	58.0
Sex2_S5	1,007,577	0	61.7	18–26	57.0
Sex3_S6	1,169,125	0	62.9	18–26	57.0

Numbers reflect the structure of the data set produced after sequence quality controls and adapter removal

As no reference genome is available for *P. notatum*, small RNA reads were mapped onto a *Paspalum* reference transcriptome generated using RNA samples extracted from developing spikelets (premeiosis, meiosis, postmeiosis, anthesis) collected from the two genotypes used in this work (Q4117 and C4-4x) [24]. In particular, this reference was assembled using 454/Roche FLX+ sequences. It contains 48,842 isogroups (genes), out of which 18% had at least two isotigs (alleles) and was validated for ~ 100 sequences previously characterized. Based on these results, this reference likely represents a comprehensive resource that reflects transcripts variation and occurrence. Therefore, we assume that its use greatly helped to contend the constraints resulting from the polyploid and highly heterozygous nature of Q4117 and C4-4x during mapping and subsequent sRNA representation comparisons. Moreover, since both genotypes used in the analysis are tetraploid, the polyploidy status should not interfere with results. Only 21–23% of the reads could be mapped using the Bowtie aligner, indicating that a large fraction of the sRNAs derived from sequences with low expression in flowers or corresponded to non-silencing small RNAs (Table 2). The average numbers of mapped small RNA reads on apomictic and sexual libraries were equivalent (216,543.66 ± 53,362.90 and 207,796.00 ± 18,421.28, respectively; *t* = 0.831, paired samples) and clustering analysis revealed high similarities between replicates (Fig. 2 b). Out of 67,617 reference transcripts (assembled into 48,769 genes), 12,942 (19.14%) were targeted by small RNA sequences (*n* ≥ 5.0 considering all libraries) and thus selected for further analyses. The number of reads mapped in this set of transcripts for each library is provided (Additional file 3, section 1) together with the list of the top 1000 transcripts showing the highest read coverage (Additional file 3, section 2). Within the most represented targets, clustering revealed differences in the number of mapped reads occurring in both directions (*i. e.* a significant higher number of sRNA reads in the apomictic or the sexual libraries) (Fig. 2 c).

**Table 2:** Mapping of sRNA reads from apomictic and sexual libraries onto the *Paspalum* transcriptome reference [24]

Sample name	% aligned	Millions of reads aligned
Apo1_S1	22.6	0.3
Apo2_S2	22.4	0.2
Apo3_S3	22.6	0.2
Sex1_S4	22.9	0.3
Sex2_S5	21.0	0.2
Sex3_S6	21.7	0.3

Many genes involved in reproduction have been identified in the *Paspalum* reference transcriptome. Next, we focused in determining to which extent small-RNA target genes belong to this particular group. To achieve this, we first categorized *O. sativa* orthologs of annotated small RNA target genes (4560 unique genes) using the plant structure development stage ontologies provided Plant Ontology database [45]. This revealed numerous genes with ontologies associated with male and female reproduction (e.g. androecium development stage, gametophyte development stage, inflorescence development stage, mature plant embryo stage, plant embryo development stage, pollen mother cell meiosis, reproductive shoot system development stage, reproductive shoot system development stage, and sporophyte reproductive stage) (Additional file 3, section 3). Considering GO ontologies, only a few families were highly represented in the *Paspalum* reference transcriptome: within the class ‘biological processes’, the subclasses ‘cellular process’ (GO: 0009987) and ‘metabolic process’ (GO: 0008152) resulted predominant, representing 29.3 and 28.8% of the transcripts, respectively; within the class ‘cellular component’, the most represented subclasses were ‘cell part’ (GO: 0044464) and ‘organelle/nucleus’ (GO: 0043226), with 39.6 and 29.1% of the transcripts, respectively. Moreover, within the class molecular function, the subclasses ‘catabolic activity’ (GO: 0003824) and ‘binding’ (GO: 0005488) reached 50 and 27.8% of the total transcripts, respectively (Additional file 3, section 4, panel A). Analysis for overrepresentation in target genes compared to the full floral transcriptome showed significant enrichment for these families or related subfamilies (Additional file 3, section 4, panel B).

Next, edgeR was used to compare the number of sRNA reads between sexual and apomictic libraries that map over all *Paspalum* floral transcripts. Counts and edgeR scores are detailed in Additional file 3, section 5, and pictured in Fig. 2 d. Using an FDR cut-off value of 0.05, we identified 1525 transcripts showing significant differences. These transcripts split evenly into two groups, including those that match sRNAs overrepresented in apomictic samples (753) or in sexual samples (772). However, if we consider only those transcripts displaying larger differences (> four-fold change), a higher number match sRNAs overrepresented in apomictic samples compared to sexual ones (480 vs 384; *p* = 0.0014, Pearson’s chi-squared test). Interestingly, among all differential transcripts, we detected several homologs of *O. sativa* genes encoding proteins involved in: 1) meiosis: MEIOSIS-SPECIFIC DNA RECOMBINASE, SYNAPTONEMAL COMPLEX (SC) ASSEMBLY; MALE-GAMETE-SPECIFIC HISTONE H3; 2) biomolecules transport: AMINO ACID/AUXIN PERMEASE 15; MULTIDRUG RESISTANCE 9; MRS2/MGT FAMILY MEMBER 9; ABC TRANSPORTER I FAMILY MEMBER 8; MONOSACCHARIDE TRANSPORTER 1; SORBITOL TRANSPORTER; AMMONIUM TRANSPORTER 3 MEMBER 1; METAL TOLERANCE PROTEIN 8.1; HIGH-AFFINITY POTASSIUM(K+) TRANSPORTER 13; AMINO ACID TRANSPORTER-LIKE 5; and BI-DIRECTIONAL AMINO ACID TRANSPORTER 1; 3) hormone signaling pathways: AMINO ACID/AUXIN PERMEASE 15; MITOGEN-ACTIVATED PROTEIN KINASE KINASE KINASE; GIBBERELLIN-INSENSITIVE DWARF 2; INDOLE-3-ACETIC ACID (IAA)-AMIDO SYNTHETASE GH3–8; and IAA30; 4) transcriptional regulation: b-ZIP TRANSCRIPTION FACTOR 71; ETHYLENE RESPONSE FACTOR 77 (APETALA-2-LIKE TRANSCRIPTION FACTOR); FINGER PROTEIN WZF1; WRKY GENE 67; BEL1-LIKE HOMEODOMAIN GENE 6; MADS27; and GROWTH-REGULATING FACTOR 1, 3, 9, and 10; and 5) cell cycle and proliferation: CELL DIVISION CYCLE PROTEIN 20.1; and CELL DIVISION CONTROL PROTEIN 2 HOMOLOG 2 (Additional file 3, section 5). In summary, mapping small RNA reads onto the *Paspalum* reference transcriptome revealed that numerous transcripts homologous to *O. sativa* genes associated with plant reproductive development, plant hormone (auxin/cytokinin) signaling, biomolecules transport, transcription control and cell cycle undergo differential epigenetic regulation during apomictic and sexual reproductive development.

Previous comparative transcriptome analyses of apomictic and sexual *P. notatum* floral reference libraries (454/Roche FLX +) revealed a list of 3732 (out of 67,617) isotigs associated with apomixis [24], which indicated that global differential expression comprises 5.51% of the total transcripts. Here, we found 1525 transcripts displaying differential sRNA representation, which accounts for 2.25% of the total 67,617 reference library isotigs. According to these data, apparently 40.8% of differential expression could be assigned to the occurrence of silencing events. However, a correlation between the two comparisons (differential sRNA and long transcript representation in apomictic vs. sexual samples) is not strictly linear, because they were originated from different experimental designs. There is a contrasting output capacity inherent to both techniques: while Illumina produces massive quantity of short reads, 454/Roche FLX + generates longer reads, but many of them are represented by a low number. High output facilitates the discovery of statistically significant differential representation, because of the higher sample size (n) used in comparisons, which might lead to a consequent overestimation of the percentage of differentially expressed genes being targeted by silencing mechanisms. An accurate estimation of the percentage of differentially expressed genes subjected to silencing would require the mapping of sRNA reads onto floral Illumina reference libraries, which are currently not available for *P. notatum*. Besides, since our libraries originated from bulks, our approach did not allow to discriminate at which specific stage differential expression occurred. Nevertheless, we likely detected differential expression across all developmental stages and further investigations such as in situ hybridization or qPCR experiments should help in resolving temporal differences.

### Discovery of *Paspalum* miRNA genes

**Fig. 3:**
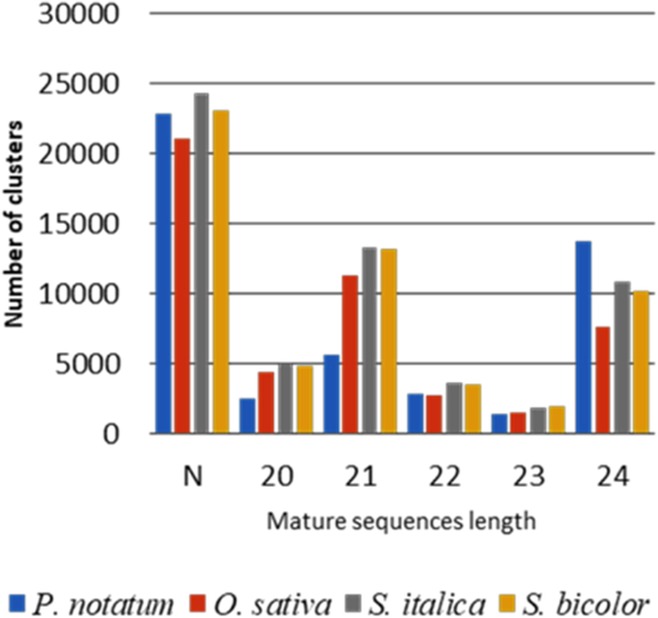
Histogram of length distribution of sRNA sequences. Sequences were associated to predicted clusters using four references. N: lengths inconsistent with DICER activities

To search for miRNAs expressed during reproductive development in *P. notatum*, we computed small RNA sequences with ShortStack using the *Paspalum* floral transcriptome and the closely related genomic sequences of *O. sativa*, *Setaria italica* and *Sorghum bicolor* as references. Percentages of unmapped reads varied from 58.0% in *S. bicolor* to 69.7% in *P. notatum* (Table 3). Among mapped reads, the highest proportion of uniquely mapped sequences was detected for the *P. notatum* transcriptome (16.7%), while it dropped down to 8.0% for heterologous genomic references (Table 3). This result was anticipated, since the *Paspalum* transcriptome reference displays less sequence variability and redundancy than heterologous genomic references. We detected a maximum of small RNA clusters in *S*. *italic* and a minimum in rice (Table 3), most of them producing DicerCall scores (i.e. mature small-RNAs lengths) inconsistent with Dicer endoribonuclease activity (Fig. 3). Interestingly, ShortStack analysis revealed no evidence for the occurrence of phased small RNAs (PhaseScore < 10 for 21 and 24-nt reads) across the four references used.

**Table 3:** Summary of primary floral *P. notatum* sRNA alignment over four references using ShortStack v.5

Reads alignments	References			
	*P. notatum* transcriptome	*O. sativa* genome	*S. bicolor* genome	*S. italica* genome
Uniquely mapped (%)	16.4	6.5	8.0	7.4
Multi-mapped (%)	13.9	23.9	34.0	31.9
Unmapped (%)	69.7	69.6	58.0	60.7
Numbers of clusters	49,1	48,8	56,9	58,8
Number of miRNA precursors	8	35	42	42

Computational analysis for hairpin configuration detected 8, 35, 42 and 42 putative miRNA precursors over *P. notatum, O. sativa*, *S. italica* and *S. bicolor* references, respectively (Table 3). Candidate miRNA genes mainly generated mature miRNA sequences of 21–22 nt (Fig. 3). The corresponding transcripts of *P. notatum* and the genomic locations in reference genomes of all precursors, as well as, the length and the sequence of the major mature miRNAs detected are shown in Additional file 4, section 1. Precursor coverage ranged from 3 to 221,108 short reads, and from 2 to 213,532 for major mature sequences. Three candidate clusters were eliminated for further analyses due to low read coverage (n ≤ 5). The resulting 124 miRNA precursors distributed over all chromosomes of the three genomic references but rice chromosome 11, *S. bicolor* chromosome 5, and *S. italica* chromosome 8 (Additional file 4, section 1). Interestingly, two putative miRNA precursors detected using the rice genomic reference (Cluster_9305 and Cluster_9306) mapped within a region of chromosome 12 previously identified as syntenic to the region associated with apomixis in several *Paspalum* species [42, 46, 47].

### Identification of conserved and novel miRNAs expressed in the floral transcriptome of *P. notatum*

To identify conserved and novel miRNAs expressed in the floral transcriptomes of sexual and apomictic *P. notatum,* mature miRNA sequences were searched in MirBase [48] using the BLAST SSEARCH option and an E-value < 0.05. The 124 predicted sequences generated 40 unique conserved miRNA accessions, corresponding to 22 miRNA families, including miR156, miR166, miR164, miR167, miR171, miR396, and miR2275 as the most abundant ones (Table 4; Additional file 4, section 2). Most families had accessions detected in three out of the four references (*n* = 17), but three of them (miR167, miR396 and miR2275) were found across all four references (Table 4).

**Table 4:** Unique miRNA mature sequences present on the floral transcriptome of sexual and apomictic *P. notatum*

miRNA mature sequence	nt	Accession*	Pn	Os	Sb	Si	Family
GCUCACUUCUCUUCCUGUCAGC	22	MIMAT0015124	0	0	0	1	miR156
GCUCACUUCUCUCUCUGUCAGU	22	MIMAT0015129	0	0	1	0	miR156
GCUCACUGCUCUGUCUGUCAUC	22	MIMAT0015128	0	1	1	1	miR156
UUGACAGAAGAGAGUGAGCAC	21	MIMAT0026186	0	1	0	0	miR156
UUUGGAUUGAAGGGAGCUCUG	21	MIMAT0001023	0	1	1	1	miR159
UGCCUGGCUCCCUGUAUGCCA	21	MIMAT0000178	1	0	2	2	miR160
UGGAGAAGCAGGGCACGUGCA	21	MIMAT0000185	0	1	1	1	miR164
UGGAGAAGCAGGGCACGUGCU	21	MIMAT0001034	0	1	1	1	miR164
CAUGUGCCCAUCUUCUCCACC	21	MIMAT0015138	0	1	0	0	miR164
CACGUGCUCCCCUUCUCCACC	21	MIMAT0015319	0	1	0	0	miR164
UCGGACCAGGCUUCAUUCCUC	21	MATAT0001072	0	1	1	1	miR166
UCGGACCAGGCUUCAUUCCCC	21	MIMAT0000189	0	5	5	5	miR166
UCGGACCAGGCUUCAAUCCCU	21	MIMAT0001037	0	1	2	2	miR166
UGAAGCUGCCAGCAUGAUCUGA	22	MIMAT0014072	1	1	1	0	miR167
UGAAGCUGCCAGCAUGAUCUA	21	MIMAT0000196	0	1	1	2	miR167
UCGCUUGGUGCAGAUCGGGACC	22	MIMAT0001045	0	1	1	1	miR168
UGGGCGGUCACCUUGGCUAGC	21	MIMAT0026431	0	0	1	1	miR169
UUGAGCCGCGUCAAUAUCUCC	21	MIMAT0014082	0	1	1	1	miR171
GGAUAUUGGUGCGGUUCAAUC	21	MIMAT0022877	0	1	0	0	miR171
GUGGUAUUGUUCCGGCUCAUG	21	MIMAT0037190	0	1	1	1	miR171
UUGGACUGAAGGGUGCUCCCU	21	MIMAT0020761	0	1	1	1	miR319
AAGCUCAGGAGGGAUAGCGCC	21	MIMAT0000931	0	1	0	0	miR390
UCCAAAGGGAUCGCAUUGAUC	21	MIMAT0000957	1	1	1	0	miR393
GUUCUCCUCAAGCACUUCACA	21	MIMAT0015347	0	1	0	1	miR395
UCCACAGGCUUUCUUGAACUG	21	MIMAT0001601	0	0	0	1	miR396
UUCCACAGCUUUCUUGAACUG	21	MIMAT0000944	2	1	1	1	miR396
UUCCACAGCUUUCUUGAACUU	21	MIMAT0000945	0	1	0	1	miR396
UUGAGUGCAGCGUUGAUGAGC	21	MIMAT0025996	0	0	1	1	miR397
UGUGUUCUCAGGUCGCCCCCG	21	MIMAT0014020	0	0	1	0	miR398
UGCCAAAGGAGAGUUGCCCUG	21	MIMAT0000952	0	0	1	1	miR399
UGCCAAAGGAGAGCUGCCCUG	21	MIMAT0000992	0	1	1	1	miR399
UGGAAGGGGCAUGCAGAGGAG	21	MIMAT0014028	0	1	1	1	miR528
AGAAGAGAGAGAGUACAGCCU	21	MIMAT0014030	0	1	1	1	miR529
UGGGUGUCAUCUCUCCUGGAGC	22	MIMAT0015366	0	0	1	0	miR1432
UUCCCGAUGCCUCCCAUUCCUA	22	MIMAT0011741	0	0	1	0	miR2118
UUCCUGAUGCCUCCCAUUCCUA	22	MIMAT0011745	0	0	0	1	miR2118
UUUGGUUUCCUCCAAUGUCUCA	22	MIMAT0035533	0	0	0	1	miR2275
CUUGUUUUCCUCCAAUGUCUCA	22	MIMAT0037125	0	1	0	1	miR2275
CUUGGUUCCCUCCAAUAUCUCA	22	MIMAT0011758	1	0	0	0	miR2275
AGGAUUAGAGGGAACUGAACC	21	MIMAT0011769	0	1	1	1	miR2275
CUCUCGCCGGCGUGCGCACUCC	22	no hit	0	0	1	0	Pn_miR1
AGUGCGCCGCCGUCGAUCUGC	21	no hit	1	0	0	0	Pn_miR2
UUGACAGAAGAGAGCGAGCAC	21	no hit	0	0	1	1	Pn_miR3
UGACAGAAGAGAGUGAGCAC	20	no hit	1	0	2	1	Pn_miR4
UGACAGAAGAGAGAGAGCAC	20	no hit	0	0	1	1	Pn_miR5
GGGCAAAUCAUCUGGGCUACC	21	no hit	0	0	0	1	Pn_miR6
UGAGCCGAGCCAAUAUCACUUC	22	no hit	0	1	1	1	Pn_miR7
UAUUGUCUCGGCUCACUCAGA	21	no hit	0	1	2	2	Pn_miR8

Sequences were detected by using four references: Pn: *Paspalum* transcriptome; Os: *Oryza sativa* Japonica genome; Sb: *Sorghum bicolor* genome; Si: *Setaria italica* genome

*Accession number was obtained by performing BLASTn analysis (SSEARCH option) of mature sequences on miRBase [48]

In addition, eight putative new mature miRNA sequences (i.e. with no significant match in MirBase) were detected (Table 4). Folding analysis of their putative precursors (named Pn_miR1 to Pn_miR8) revealed that all hairpin structures with the Dicer cleavage sites within the arm of the stem regions displayed the expected match between miRNAs and opposite miRNAs* and exhibited minimum free energy < 60 kcal. Mol^− 1^ (Additional file 5). Thus, these 8 miRNA mature sequences were considered as novel plant miRNAs. Precursors of Pn_miR1, Pn_miR3, Pn_miR5, Pn_miR6, Pn_miR7 and Pn_miR8 were found using genome references only. Pn_miR4 was detected using the *P. notatum* transcriptome and the *S. bicolor* and *S. italic* genomes. In contrast, Pn_miR2 was found using the *P. notatum* transcriptome only, which suggests that it might be specific to *P. notatum*. Finally, a phylogenetic analysis considering all unique mature miRNA sequences detected in our analysis showed that Pn_miR3, Pn_miR4 and Pn_miR5 are evolutionarily related to miR156, while Pn_miR7 is related to miR171 (Additional file 6). Association of the remaining four Pn_miRNAs with conserved accessions could not be supported by the bootstrapping analysis (< 65%), suggesting they might be novel plant miRNAs.

### Differentially expressed miRNA genes

**Fig. 4:**
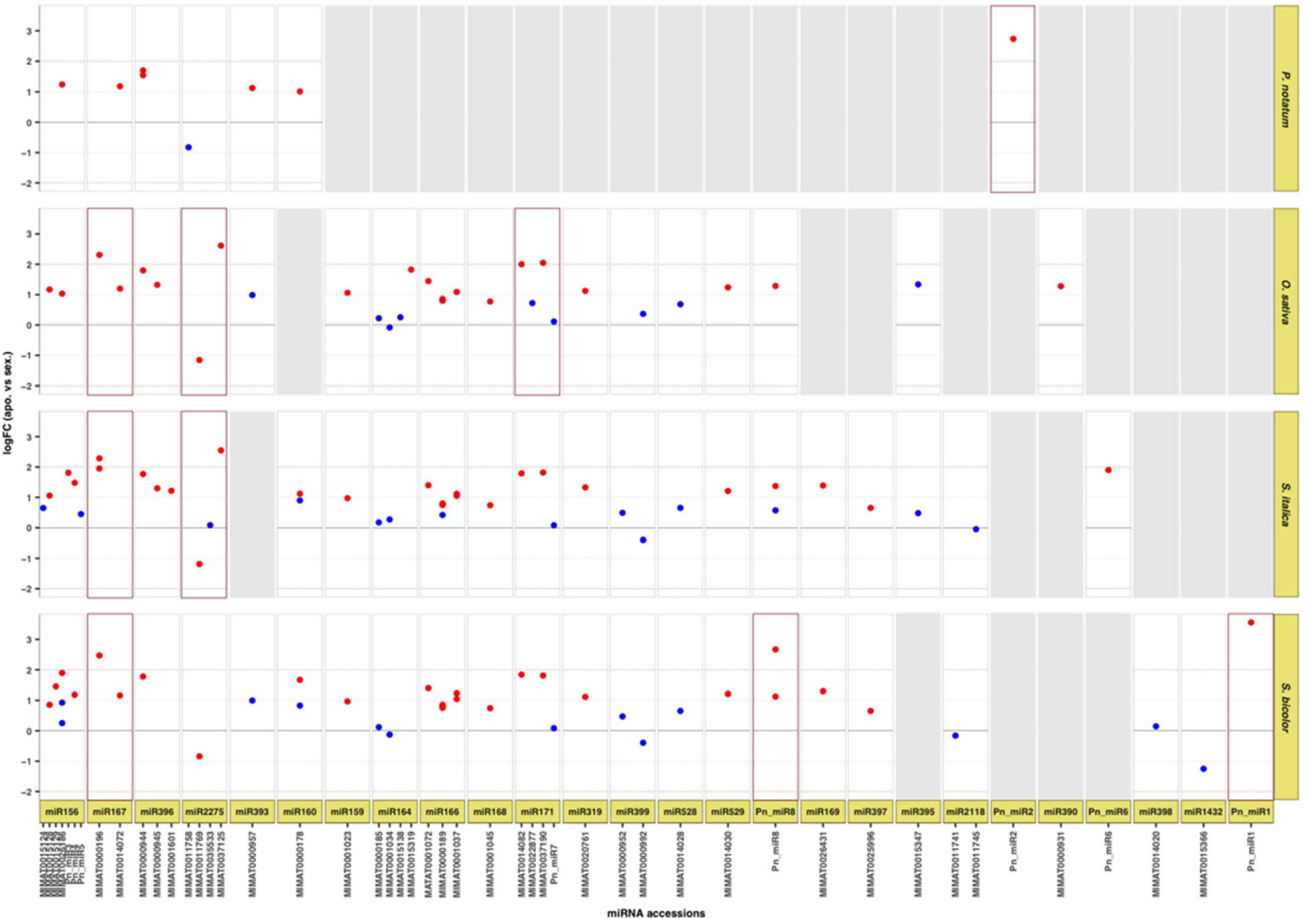
Differential expression of predicted miRNA genes. Panels summarize coverage comparative analysis for predicted miRNA clusters ordered by miRNA families and members (*x-axis*). The references used were indicated on the right side of the panel series. Each dot indicates significance (red: FDR < 0.05; blue: FDR > 0.05). The log2 fold change (*y-axis*) was obtained from the edgeR analysis. miRNA members are indicated by using miRbase [48] annotations for conserved mature miRNAs. Magenta-boxed panels display miRNA families with at least one predicted cluster with more than four-fold expression change. Grey boxes: no cluster predicted

Small RNA read counts derived from both apomictic and sexual libraries were normalized and analyzed for differential expression using edgeR. Out of the 124 candidate miRNA precursors, 9 showed highly significant differences between reproductive modes, being all of them over-represented in apomictic libraries (logFC ≥2.0; FDR < 0.05). According to our annotation, these loci produce both conserved (miR167/MIMAT0000196: Cluster_5800, _9305 and _20699; miR171/MIMAT0037190: Cluster_29775; miR2275/MIMAT0037125: Cluster_43159 and _34865) and novel (Pn_miR1: Cluster_3423; Pn-miR2: Cluster_13846; Pn-miR8: Cluster_13322) miRNAs (Additional file 4, section 3). In addition, we identified 56 clusters with moderate but significant differences in coverage (1 < logFC < 2, FDR < 0.05) corresponding to various families, including: miR156, miR159, miR160, miR164, miR167, miR169, miR171, miR319, miR390, miR393, miR396, miR529, and Pn_miR6. Again, most precursors showed increased expression in apomict libraries except for three clusters all containing a miR2275 accession (Additional file 4, section 3; Fig. 4).

Several of the miRNAs showing strong and modest differences in abundance between apomictic and sexual plants have been associated with many aspects of plant reproductive development, including: miR156, miR159, miR160, miR167, miR169, miR319, miR390, miR2118, and miR2275 [49–51]. Of interest, in our analysis, ath-miR167a-5p precursors detected using the three genome references were overexpressed in apomictic samples while precursors for a second member of the miR167 family (ccl-miR167a) showed a modest increase using the *P. notatum* reference transcriptome and two of the genomic references (Additional file 4, section 3; Fig. 4). Thus, miR167 precursors, two of which mapped on a rice segment of chromosome 12 syntenic to the apomixis controlling locus in *P. notatum*, appeared consistently upregulated in apomictic libraries compared to sexual ones. Alike miR167 precursors, most of the remaining families (for instance miR156, miR160, miR166, miR171, miR319, miR396, miR529), exhibited congruent expression patterns among the various precursors detected across the references used. However, the four precursors we identified for the miR2275 family showed contrasting behavior: while no significant change was detected for osa-miR2275a and bdi-miR2275a (specifically detected in *P. notatum* and *S. italica*, respectively), the two remaining precursors showed opposite changes, i.e. increased expression in apomictic libraries for ata-miR2275b-3p (for both *O. sativa* and *S. italica* references) and slightly decreased expression in apomictic libraries for zma-miR2275b-5p (for both *S. italica* and *S. bicolor* references).

### Prediction of miRNA target genes and putative miRNA-dependent pathways for installing apomixis

**Fig. 5:**
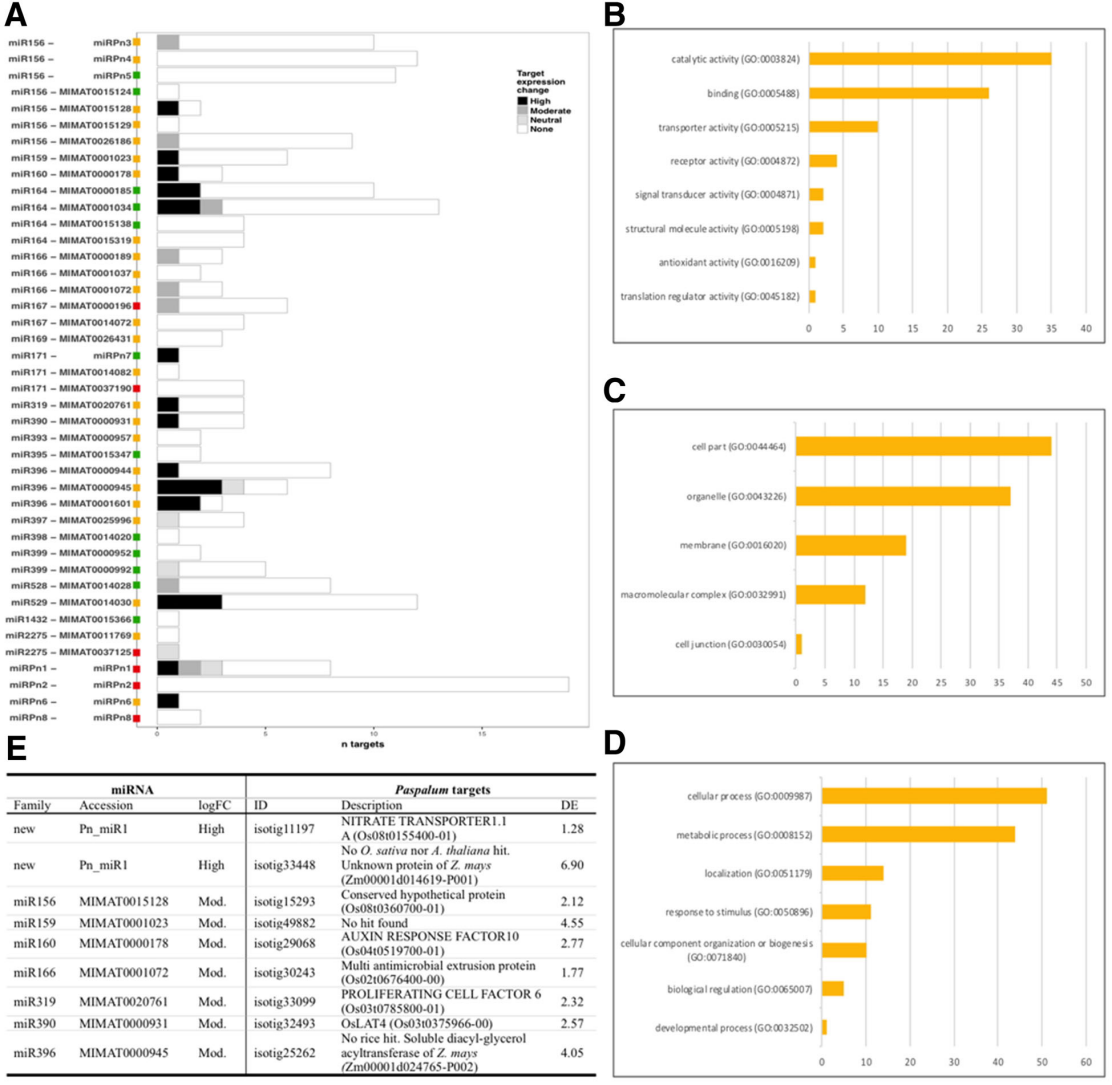
Candidate miRNA-regulated genes expressed during *P. notatum* reproductive development. **a.** Number of target genes by miRNA families. Colours in bars indicate the number of targets showing high, moderate, neutral and no change (see insert box for legend). Square boxes at the left indicate the differential expression of miRNA precursors between apomictic and sexual sRNA libraries: green: no change; orange: moderate change (1 < logFC < 2); red: high change (logFC > 2). **b-d** Significantly enriched GO classes corresponding to Molecular Function (**b**), Cell Component (**c**) and Biological Process (**d**). **f** Candidate target-miRNA regulatory modules for licensing apomixis

With the aim of identifying putative miRNAs targets*,* we computationally predicted binding sites for the 40 mature miRNAs reported in Table 4 in the *Paspalum* transcriptome reference using TargetFinder. Using a cut off value of 4.0, 376 hits were identified representing 283 different transcripts, out of which 224 were unique hits (i.e. target of a single miRNA), while 40, 11, 5, and 3 were found 2, 3, 4 and 5 times, respectively (Additional file 7, section 1). Most multiple-hits transcripts (*n* = 48) were targeted by miRNAs from a single family, but a few ones were targeted by members of two families (miRNA156/miR529; miR159/miR319; miR160/miR166; miR396/Pn_miR1, and; Pn_miR1/Pn_miR2). We identified targets for all predicted miRNA families but miR158. The number of targets varied greatly across families (from 1 to 78 for miR156) and accessions (from 1 to 19 for Pn_miR2) (Fig. 5 a). BLASTX against rice reference peptides produced an annotation for 275 (73.3%) of the 374 transcripts, representing 195 unique genes (Additional file 7, section 1). Target annotations were consistent with previous reports for target-miRNA regulatory modules, as illustrated by the occurrence of homologies to transcription factors of the *SQUAMOSA PROMOTER BINDING PROTEIN LIKE* (*SPL*) family (miR156), the *NAC* (*NAM, ATAF1/2, and CUC2*) family (miR164), the *HD-ZIP* family (miR166), the *GROWTH-REGULATING FACTOR* family (miR396), and the *AUXIN RESPONSE FACTOR* (ARF) family (miR160, miR167) [52]. The most represented GO annotations for the 196 *O. sativa* orthologs corresponding to molecular function (MF), cellular components (CC), and biological processes (BP) are shown in Fig. 5 b, c and d, respectively. For MF ontologies (60 hits), genes categorized into catalytic activity (43.2%) and binding (32.1%) (Fig. 5 b). Within the CC group (73 hits; Fig. 5b), genes associated with cell parts (39.9%) and organelles (32.7%) were the most abundant (Fig. 5 c). Likewise, within the BP class (103 hits) cellular processes and metabolic processes (37.5 and 32.4%, respectively) were also highly represented (Fig. 5 d).

Next, to identify putative major molecular routes differentially modulated by miRNAs during sexual and apomictic developments, we mined the *Paspalum* reference transcriptome for candidate target transcripts already assessed for differential expression between apomixis and sexuality [24]. Out of 374 predicted target transcripts, 207 could be analyzed (n reads > 10 in at least one sample) and a significant difference (FDR < 0.05) was found for 34 transcripts (17.6%) targeted by members of 14 miRNA families. As shown in Fig. 5 a, high fold changes prevailed (*n* = 21; FC > 4) over moderate (*n* = 6; 1 < FC < 2) and low (*n* = 5; FC < 1) differences. However, despite increased expression in apomictic libraries affected most miRNA genes differentially expressed between apomictic and sexual libraries, no bias in direction change was observed for the predicted targets (18 downregulated vs. 16 upregulated).

Then, miRNA/target pairs with consistent opposed expression were identified by comparing changes in representation for miRNAs and their target transcripts (Additional file 7, section 2). Considering logFC > 2, we detected one miRNA displaying an increased expression congruent with a transcriptional decrease of its targets: Pn_miR1 (targets: isotig11197and isotig33448, encoding NITRATE TRANSPORTER1.1A and the unknown protein Zm00001d014619-T001, respectively) (Fig. 5 e). Nevertheless, as the regulatory impact of miRNAs does not only depend on their expression levels, but also on their target mRNA configuration [53], we also considered targets of miRNAs whose precursors showed moderate changes (1 < logFC = < 2, FDR < 0.05). By doing that, we found seven additional genes with complementary expression patterns, showing increased expression of miRNA precursors and decreased expression of the targets. Five of them encode for homologs to plant proteins with assigned functions including: the ARF10 and PROLIFERATING CELL FACTOR 6 (TEOSINTE BRANCHED1/CYCLOIDA/PROLIFERATING CELL NUCLEAR ANTIGEN FACTOR1 or TCP family) transcription factors, and three proteins related to cellular activity (OsLAT4, Zm00001d024765-P002, and Os02t0676400–00) (Fig. 5 e). The function of the two latter ones remains unknown and one of them produced no BLASTX hit against non-redundant protein sequences (Fig. 5 e).

ARFs (auxin response factors) are key proteins involved in many developmental processes through the regulation of auxin responsive gene expression by targeting specific binding sites within their promoter [54, 55]. The current working model for auxin perception and signaling stipulates ARF inhibition at low auxin levels via dimerization with AUX/IAA and TOPLESS recruitment, whereas higher levels of auxins allows ARF activation by ubiquitinization of AUX/IAAs, which bind to the SCF^TIR/AFB^ complex [56]. Recent evidence shows that several of the numerous *ARF* genes present in the plant genomes are post-transcriptionally regulated by small RNAs; interestingly, a critical role of auxin in controlling female gametophyte differentiation was reported in *Arabidopsis* [57] and miR160 was reported as down-regulated before fertilization in rice ovules while three of its targets (OsARF10, OsARF18 and OsARF22) increase in expression during female gametophyte formation [58]. Furthermore, TCP transcription factors (including PROLIFERATING CELL FACTOR 6) are central in establishing a link between cell proliferation and hormone response, and they are also involved in many aspects of plant development, including reproduction [59, 60]. TCP6 belongs to Class I TCP transcription factors, a subfamily out of which several members have been reported to participate in auxin homeostasis in the *Arabidopsis* gynoecium by binding the *IAA3* promoter [61, 62]. In addition to these two candidate genes pointing out auxin perception and response as essential processes affected in apomictic ovules, plant nitrate transporters are known to interact with a wide range of biomolecules, including hormones, such as NITRATE TRANSPORTER 1.1 (NRT1.1), which shows affinity for IAA [63]. NRTs belong to the large PRT/NRT1 (peptide transporter/nitrate transporter 1) family of transporters that perform essential function during plant development [64]. Several PRT/NRT1 members are involved in early embryogenesis and gametophyte development in *Arabidopsis* [65, 66], including the homolog of OsNRT1.1A, whose loss of function provokes embryo arrest [67]. Furthermore, several transporter genes for different biomolecules (amino acid, sugars, ions) targeted by small RNAs also showed significant decrease in expression.

In summary, sRNA, miRNA and target analyses identified 14 molecules related with auxin transport, metabolism and response and encoded by a gene that differed in expression between sexual and apomictic plants, including: AMINO ACID/AUXIN PERMEASE 15 (auxin influx carrier) [68]; INDOLE-3-ACETIC ACID (IAA)-AMIDO SYNTHETASE GH3–8 (catalyzes the synthesis of indole-3-acetic acid (IAA)-amino acid conjugates, providing a mechanism for the plant to cope with the presence of excess auxin) [69]; IAA30 (repressor of early auxin response genes at low auxin concentrations) [70]; miR160 and miR167 (regulate expression of ARFs auxin response factors) [69]; miR164 (related with TCP-controlled auxin response) [49–51]; targets ARF2; ARF8; ARF10; ARF12 (auxin response factors) [54, 55]; AFB2 (AUXIN SIGNALING F-Box 2) [71]; PROLIFERATING CELL FACTOR 6 (TCP family) (auxin homeostasis, IAA3 promoter binding) [61, 62]; NITRATE TRANSPORTER 1.1 (NRT1.1) [63]. Moreover, a comparative analysis of the apomictic and sexual *Paspalum* floral reference transcriptomes revealed more 40 genes related to auxin metabolism and signaling showing differential expression [24]. Altogether, these findings suggests that auxin metabolism and response might be perturbed during reproductive development in *P. notatum* apomictic biotypes, resulting in altered signaling pathways and ultimately affecting growth and development.

## Conclusions

Our analysis of the small RNA expressed on the floral transcriptome of sexual and apomictic genotypes indicated that numerous genes belonging to PO ontology classes associated with reproductive development are probably controlled by epigenetic mechanisms and differentially expressed between both reproductive types. Moreover, the analysis of the small reads data set combining several Gramineae genome references and *Paspalum* transcriptomic resources allowed the detection of miRNAs from 22 known conserved plant families, predicted novel members for two conserved plant families (miR156 and miR171) and four novel Gramineae miRNA families, including one specific to *P. notatum*. Furthermore, an integration of differential representation analyses for small RNAs, miRNA genes and miRNA targets suggested that small RNA-dependent auxin-centered mechanisms might be involved in the switch from sexuality to apomixis. The differences we observed in small RNA targets and miRNA biogenesis provide novel, attractive hypotheses that will guide future functional research. With regards to this, addressing the roles of miR160, miR167 and miR319 during sexual reproduction appears critical to expand our understanding of the molecular mechanisms underlying apomixis in *Paspalum* species.

## Methods

### Plant material

Two *Paspalum notatum* tetraploid (2n = 4x = 40) genotypes were used to collect material for small RNA libraries construction: Q4117, a highly heterozygous natural accession collected from the State of Rio Grande do Sul (Southern Brazil) that reproduces through obligate aposporous apomixis [72] and C4-4x, a fully sexual genotype experimentally obtained from chromosome duplication of a highly heterozygous sexual diploid by colchicine treatment [30]. Due to the heterozygous nature of the diploid of origin, many of the C4-4x loci are expected to be heterozygous as well. However, a lower degree of heterozygosity is expected with respect to Q4117. *P. notatum* is a perennial rhizomatous species, therefore allowing multiplication through vegetative replicas. Both genotypes (Q4117 and C4-4x) belong to the living *Paspalum* germplasm collection established at IBONE-CONICET-UNNE (Instituto de Botánica del Nordeste, Corrientes, Argentina). Vegetative replicates were transferred and maintained in experimental plots at IICAR-CONICET-UNR (Instituto de Investigaciones en Ciencias Agrarias de Rosario, Zavalla, Argentina). Replicates of both Q4117 and C4-4x were established in neighboring plots in order to guarantee identical growing environmental conditions.

### Library preparation, sequencing and quality control

Small RNAs libraries were produced in triplicate from evenly balanced bulks (*i. e.* balanced mixes) of spikelets collected at four developmental stages: premeiosis, meiosis, postmeiosis and anthesis. The material was classified into different developmental stages categories by correlating micro- and megagametophyte development and following the *P. notatum* reproductive calendar, as recommended in previous work [20]. Equivalent amounts (mg) of plant material (spikelets) from each developmental stage were mixed in order to produce two bulks (apomictic and sexual), whose comparison is expected to reveal contrasts between reproductive modes, yet it will not provide temporal resolution. Briefly, total RNA was extracted using the SV Total RNA Isolation System (Promega). RNA samples were then quantified by fluorescence (Qubit 2.0 Fluorometer, Thermo Fisher Scientific) and RNA integrity was determined using an Agilent Bioanalyzer 2100. Small RNA libraries were prepared following the Illumina TruSeq Small RNA sample preparation guide and 50-bp single-end sequencing was performed using the Illumina MiSeq system by Polo d’Innovazione di Genomica, Genetica e Biologia (Polo GGB), Siena, Italy. Raw reads were trimmed for adapters and low sequence quality using Cutadapt v1.3 [73], FastQC v.0.11.5 [74] and multiQC [75]. Sequences were finally filtered for 18-26 nt length and for Phred quality score > 25. The sRNA datasets were deposited in the NCBI Sequence Read Archive (SRA) repository under accession number SRP099144.

### *Paspalum* transcripts annotation and AGO proteins phylogenetic analysis

We re-annotated the *Paspalum* transcriptome reference by comparing transcripts with the *Oryza sativa* Japonica reference peptides (genome assembly IRGPS-1.0) provided by the Gramene database [76]. For global annotation, we retained Blastx best alignments with an e-value < 0.05 and an identity of at least 50%, resulting in 33,071 annotated isogroups/genes (out of 51,168). More stringent criteria were used for searching genes with annotations related to small RNA-directed pathways (e-value <1e^− 05^, ID > 50%, and alignment length > 200). For AGO proteins phylogenetic analysis, we used the MUSCLE alignment and the Neighbor-joining options of Geneious®10.0.9. Phylogenetic tree was inferred after careful visual inspection and post-processing multiple alignments for masking gaps and using bootstrap values (1000 replicates). *A. thaliana* and *O. sativa* Japonica sequences were recovered from the Gramene database [76] and *P. notatum* protein sequences were predicted from selected transcripts.

### Small RNA reads alignments and representation over the *P. notatum* transcriptome

Small RNA reads were aligned onto the 454/Roche FLX+ *P. notatum* reference floral transcriptome generated from Q4117 and C4-4x genotypes (referred to as *Paspalum* reference transcriptome) [24]. Mapping was performed using the Bowtie aligner [77] with the following parameters: no mismatch allowed (−n 0), 19 high quality bases required to initiate the alignment (−l 19) and reporting the best alignments only (‘best’ option). Read counting for each reference transcript was performed with the feature counts module of Subread [78] using Q = 10 and no strand selection. Normalization of reads counts over transcripts was carried out using the upper-quartile method option of edgeR [79]. Only transcripts showing a normalized count value per millions (cpm) of 1, in at least two samples were considered for further analyses. The edgeR package [79] was used to analyze differential expression following a glm approach [79]. Sexual samples were established as controls, therefore positive values of logFC were associated to an increased number of sRNA reads in apomictic libraries and negative values indicated an increased number of sRNA reads in sexual libraries. Read coverage comparison resulting in an adjusted *p*-value < 0.05 (FDR) [80]. Comparisons producing FDR < 0.05 and fold changes in the range of 1–2 and > 2 were considered as moderate and high coverage differences, respectively.

### Identification of conserved and novel microRNA loci expressed in the floral transcriptome of *P. notatum*

In order to predict miRNA genes expressed in the *P. notatum* floral transcriptome, small RNA sequences were analyzed with ShortStack version v05 using default parameters [81]. Since no genomic reference is available for *Paspalum*, small RNA alignments were performed using the *Paspalum* reference transcriptome and three other genomic sequences of related grasses including: *Oryza sativa* Japonica (Assembly GCA_001433935.1), *Sorghum bicolor* v2 (Assembly: GCA_000003195.2) and *Setaria italica* JGIv2.0 (Assembly GCA_000263155.2). Briefly, we aligned the small RNA reads against each reference using Bowtie allowing two mismatches (−n 2) and requiring 18 high quality bases to initiate the alignment (−l 18). Then, candidate cluster (i.e. genome/transcriptome-wide regions containing small RNAs accumulation) associated to small RNA sequences were identified with the default parameters. Putative miRNAs were then selected based on a DicerCall of 20–22. Mature miRNA sequences were compared against miRBase [48] using the SSEARCH option and a cut off E-value < 0.05 to detect conserved miRNAs. Mature sequences that produced no hit were considered as putative new *P. notatum* miRNAs (Pn_miRNAs). The secondary structure of the predicted novel miRNA genes was analyzed using RNAfold within the ViennaRNA Package [82] and manually examined for the presence of putative mature miRNA sequences in one arm of the stem region, bulges with no more than 3 unpaired nucleotides, a loop of at least 10 nucleotides in length and free-folding energy lower than or equal to − 60 kcal mol^− 1^ [83]. Multiple mature sequence alignments were performed with the software Mega6 [84]. The phylogenetic relationships among sequences were reconstructed using the UPGMA method with default parameters and assessed using 1000 bootstrap replications.

### Expression analyses of miRNA precursors

Differential expression of miRNA precursors was carried out using edgeR [79]. Only 20–24 nt reads with counts ≥5 were retained for the analysis. Read counts over precursors were normalized by library size, using the default TMM (trimmed mean of M-values) method [79]. Normalized read numbers on each precursor in apomictic and sexual samples were compared using the sexual sample as a reference (apo vs sex). Thus, positive and negative logFC values indicated higher and lower miRNAs expression levels, respectively. Sequences with corrected *p* values (FDR) ≤ 0.05 and logFC ≥ |1| were considered differentially expressed.

### Identification of miRNA target transcripts in *Paspalum* and differential expression

We used TargetFinder [85] with the default parameters and a cut off value of 4 to predict miRNAs targets expressed in the *Paspalum* reference transcriptome. Common ontologies (GO-Slim and PO) were established using the PANTHER [86] and Planteome [45]. Finally, for differential expression of *Paspalum* targets between sexual and apomictic developments, we took advantage of former expression analysis, considering only those transcripts that showed at least 10 reads in one of the libraries [24].

## Additional files

Additional file 1: Lists of annotated *Paspalum* floral transcripts involved in sRNA-directed pathways. **Section 1.** List of key rice genes involved in small RNA-directed pathways and their *P. notatum* homologs expressed in floral tissues. Section 2: Expression levels of floral *P. notatum* transcripts homologous to key genes involved in small RNA-directed pathways. (XLSX 22 kb)

Additional file 2: General statistics for the sequencing of triplicate floral sRNA libraries. The sRNA libraries were originated from apomictic (Apo) and sexual (Sex) *Paspalum notatum* genotypes. (PDF 178 kb)

Additional file 3: Lists of *P. notatum* floral transcripts harboring sRNA derived from apomictic and sexual libraries. Section 1: Number of small RNA reads from apomictic (Apo) and sexual (Sex) libraries mapping onto the *Paspalum* transcriptome reference. Section 2: List of the 1000 *P. notatum* transcripts with highest small RNA coverage. Section 3: Classification of the *Paspalum* annotated small RNA targets into ontology classes, according to Plant Structure Development Stage Ontologies (www.planteome.org). **Section 4.** Functional classification and PANTHER overrepresentation test for annotated small RNA targets. Section 5: edgeR analysis of small RNA reads mapped over the *P. notatum* reference transcriptome. (XLSX 6165 kb)

Additional file 4: Lists of miRNA detected in floral samples of sexual and apomictic *P. notatum* genotypes. **Section 1.** List of clusters harboring miRNAs detected in four references (*P. notatum* floral transcriptome, *O. sativa* genome, *S. italica* genome, *S. bicolor* genome) with ShortStack v.3.8.5. **Section 2.** BLAST SSEARCH of mature miRNAs sequences of *P. notatum* against MirBase. **Section 3**. edgeR expression analysis of clusters harbouring miRNAs detected using the above-mentioned four references. (XLSX 51 kb)

Additional file 5: Folding analysis of miRNA putative precursors. Secondary structures derived from folding 8 predicted miRNA precursors. (PDF 667 kb)

Additional file 6: Phylogenetic analysis of miRNA sequences. Phylogenetic analysis by maximum likelihood method of mature miRNA sequences expressed in the *P. notatum* floral transcriptome. (PDF 53 kb)

Additional file 7: miRNA targets prediction. Section 1: miRNA targets detected in the *Paspalum* transcriptome reference and annotated after blastx on a rice peptide database (E-val < 0.05 and % identity > 50). Section 2: miRNA targets with or without differential expression between sexual and apomictic libraries, as detected in the *Paspalum* transcriptome reference: read coverage analysis and annotation. (XLSX 68 kb)

## Data Availability

The datasets supporting the conclusions of this article are available in the NCBI Sequence Read Archive (SRA) repository under accession number SRP099144. Additional dataset(s) supporting the conclusions of this article are included within the article and its additional files. The plant materials used in this study belong to the living germplasm collection of Instituto de Botánica del Nordeste (IBONE), CONICET-UNNE, Corrientes, Argentina. Voucher specimens of this material are deposited at the Herbarium CTES-IBONE (publicly available), under deposition numbers: C4-4X (Quarin, C. L. 4260, barcode CTES0541627, cardboard No. 330064); Q4117 (Quarin, C. L. 4117, barcode CTES0541626, cardboard No. 233851).

## Abbreviations

ARF: Auxin response factor
dsRNA: Double-stranded RNA
ES: Embryo sac
FC: Fold change
FDR: False discovery rate
INDEL: Insertion/deletion
miRNA: Micro RNA
mRNA: Messenger RNA
PTGS: Post-transcriptional gene silencing
RdDM: RNA-dependent DNA methylation
RDR: RNA-dependent RNA polymerase
SNP: Single nucleotide polymorphism
sRNA: Small RNA
