# Supplementary material for: Small RNA-seq reveals novel regulatory components for apomixis in Paspalum notatum
Source: BMC Genomics. 2019 Jun 13;20:487. doi: 10.1186/s12864-019-5881-0 (PMC6567921; doi:10.1186/s12864-019-5881-0)
Supplement: Supplementary file 2 — General statistics for the sequencing of triplicate floral sRNA libraries. The sRNA libraries were originated from apomictic (Apo) and sexual (Sex) Paspalum notatum genotypes. (PDF 178 kb) [file 12864_2019_5881_MOESM2_ESM.pdf]

Additional file 2. General statistics for the sequencing of triplicate floral sRNA libraries originated from apomictic and sexual *Paspalum notatum* genotypes.

Sample Name	Total number of reads	% duplicate reads	Sequence length	% GC
**Apo1_S1**	2,996,675	74.3%	50	54%
**Apo2_S2**	1,981,542	70.8%	50	55%
**Apo3_S3**	1,558,547	69.5%	50	54%
**Sex1_S4**	1,986,075	71.3%	50	55%
**Sex2_S5**	2,032,618	72.5%	50	55%
**Sex3_S6**	2,248,170	72.5%	50	55%
