# Supplementary material for: Small RNA-seq reveals novel regulatory components for apomixis in Paspalum notatum
Source: BMC Genomics. 2019 Jun 13;20:487. doi: 10.1186/s12864-019-5881-0 (PMC6567921; doi:10.1186/s12864-019-5881-0)
Supplement: Supplementary file 5 — Folding analysis of miRNA putative precursors. Secondary structures derived from folding 8 predicted miRNA precursors. (PDF 667 kb) [file 12864_2019_5881_MOESM5_ESM.pdf]

Additional file 5 : Folding analysis of miRNA putative precursors

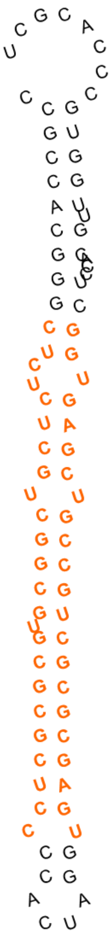

Sb cluster 3423 (Pn_miR1)

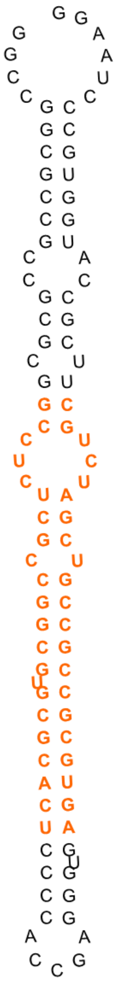

Pn cluster 13846 (Pn_miR2)

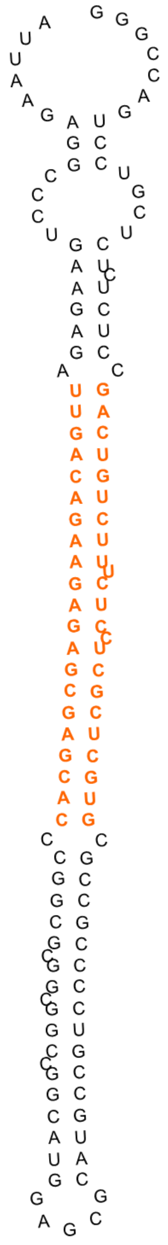

Si cluster 25130 (Pn_miR3)

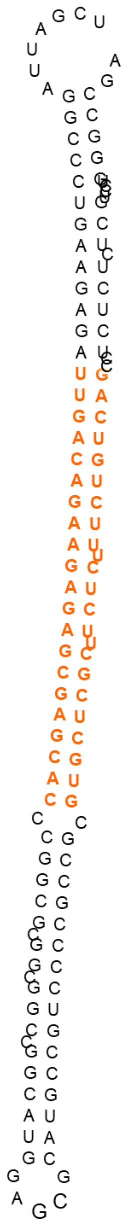

Sb cluster 4706 (Pn_miR3)

Additional file 5 (continued)

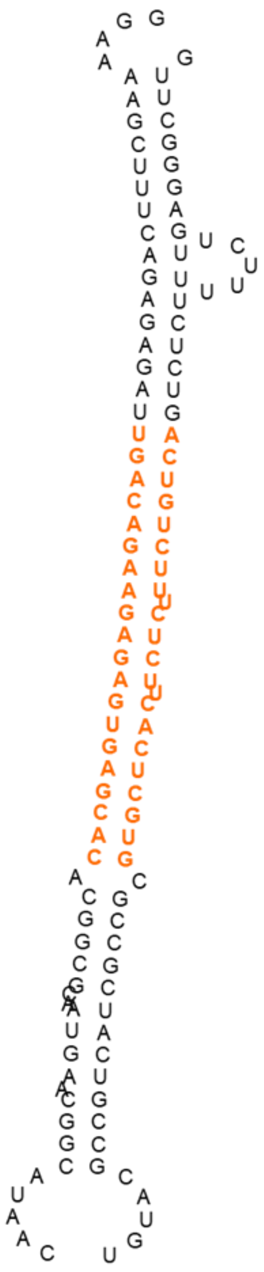

Pn cluster 5938 (Pn_miR4)

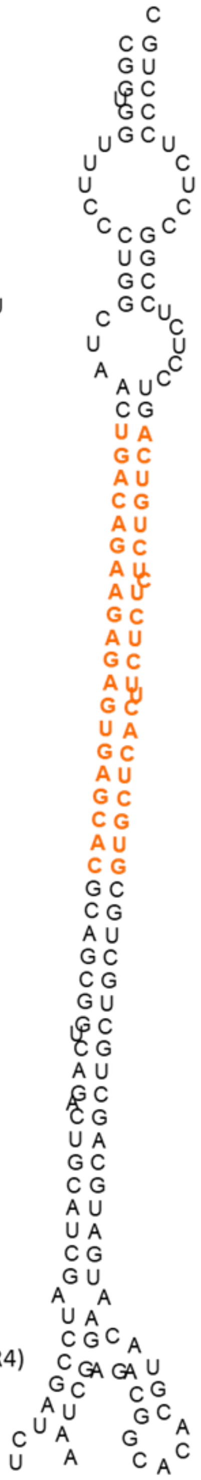

Si Cluster37461(Pn_miR4)

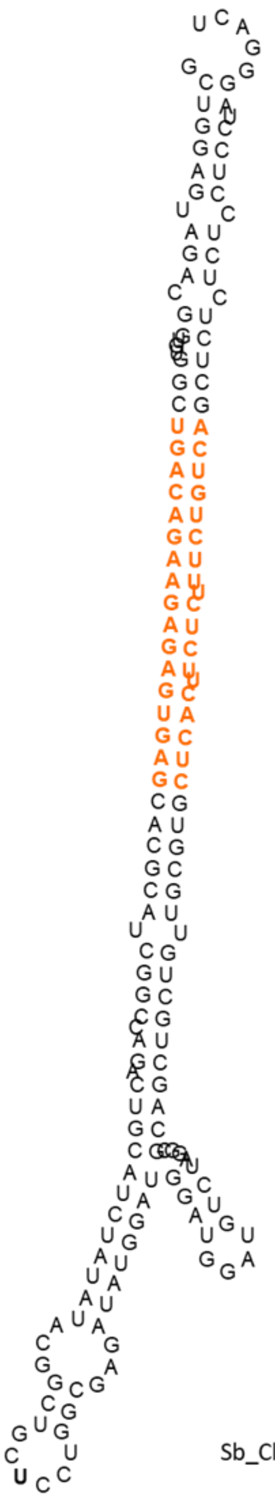

Sb_Cluster_17884 (Pn_miR4)

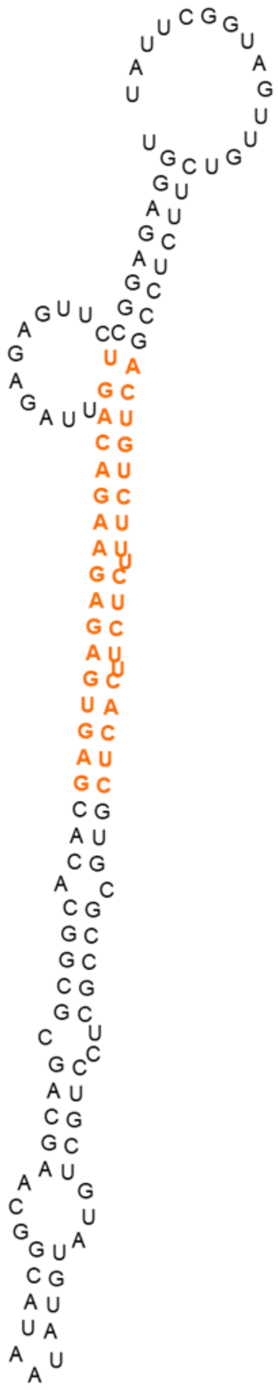

Sb_Cluster_29382 (Pn_miR4)

2

Additional file 5 (continued)

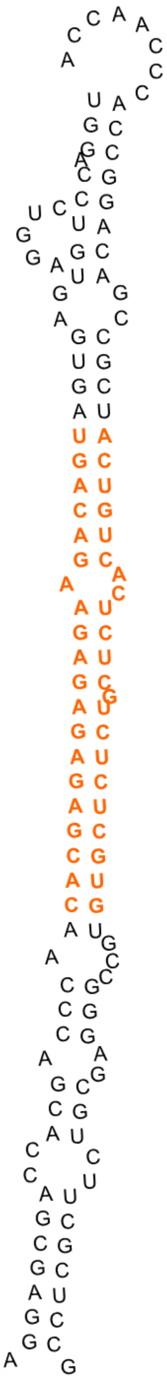

Sb_Cluster_18410 (Pn_miR5)

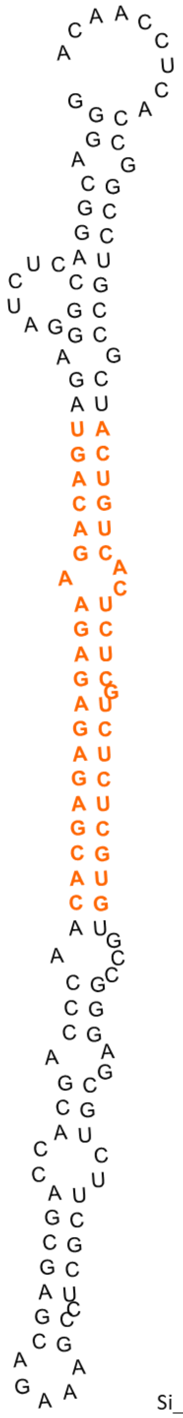

Si_Cluster_11066 (Pn_miR5)

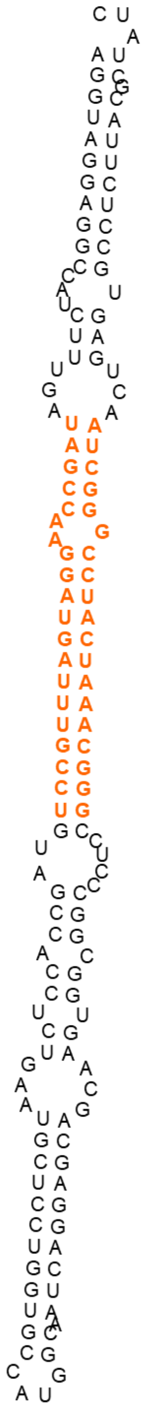

Si_Cluster_49192 (Pn_miR6)

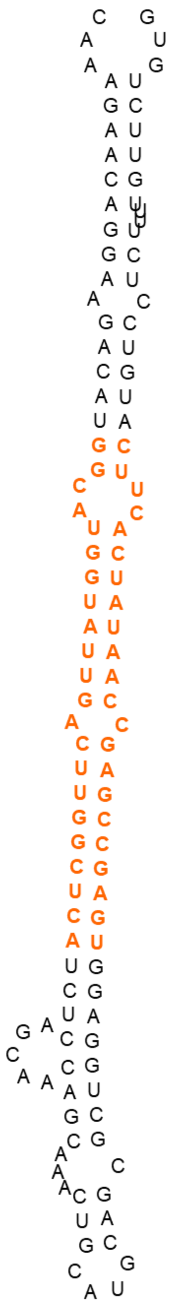

Os_Cluster_40636 (Pn_miR7)

3

Additional file 5 (continued)

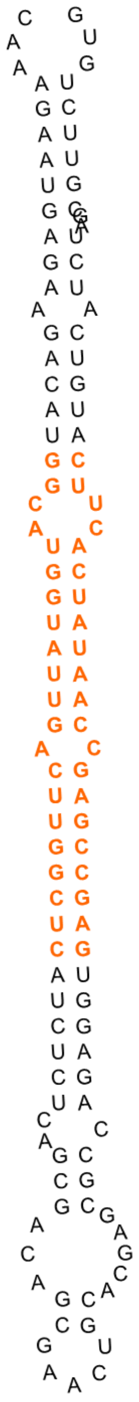

Si_Cluster_8553 (Pn_miR7)

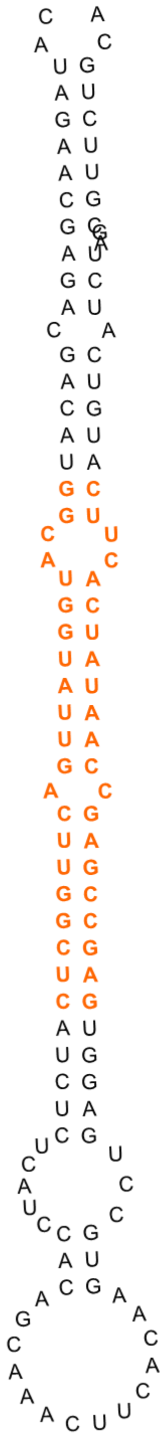

Sb_Cluster_15733 (Pn_miR7)

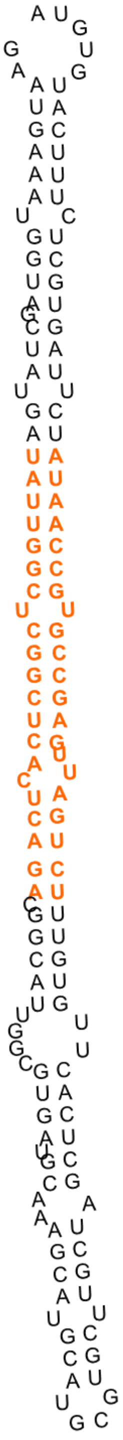

Os_Cluster_21224 (Pn_miR8)

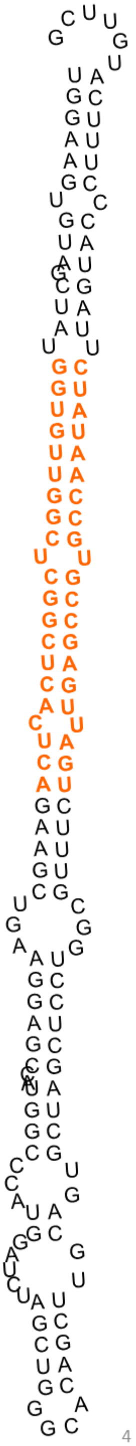

Si_Cluster_55039 (Pn_miR8)

Additional file 5 (continued)

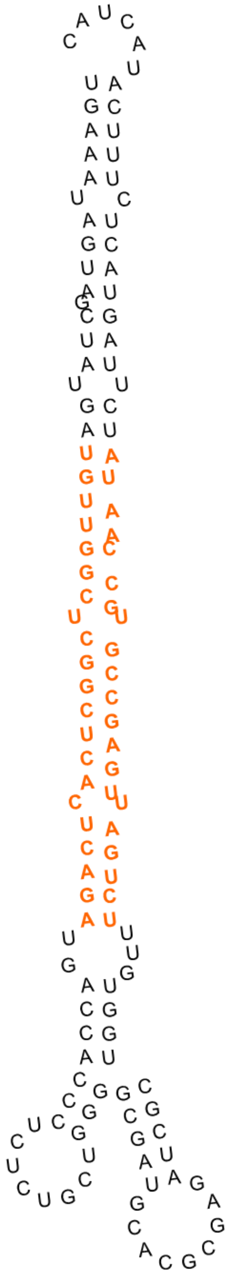

Si_Cluster_58532 (Pn_miR8)

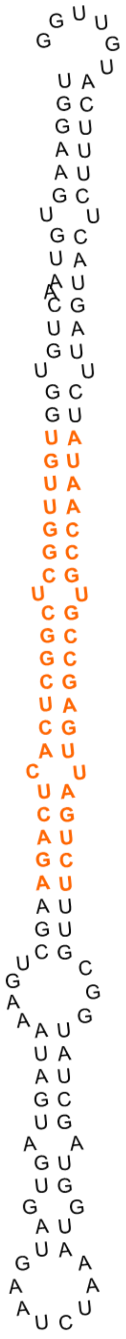

Sb_Cluster_10171 (Pn_miR8)

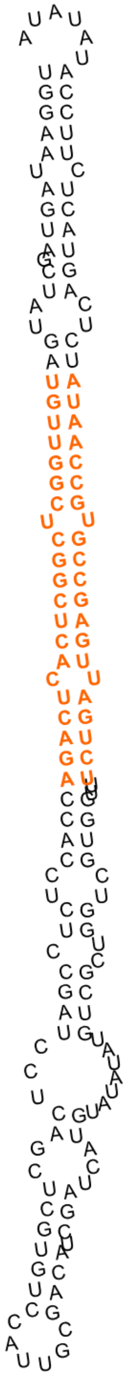

Sb_Cluster_13322 (Pn_miR8)
