# Supplementary material for: Small RNA-seq reveals novel regulatory components for apomixis in Paspalum notatum
Source: BMC Genomics. 2019 Jun 13;20:487. doi: 10.1186/s12864-019-5881-0 (PMC6567921; doi:10.1186/s12864-019-5881-0)

Additional file 6: Phylogenetic analysis of miRNA sequences. Phylogenetic analysis by maximum likelihood method of mature miRNA sequences expressed in the *P. notatum* floral transcriptome.

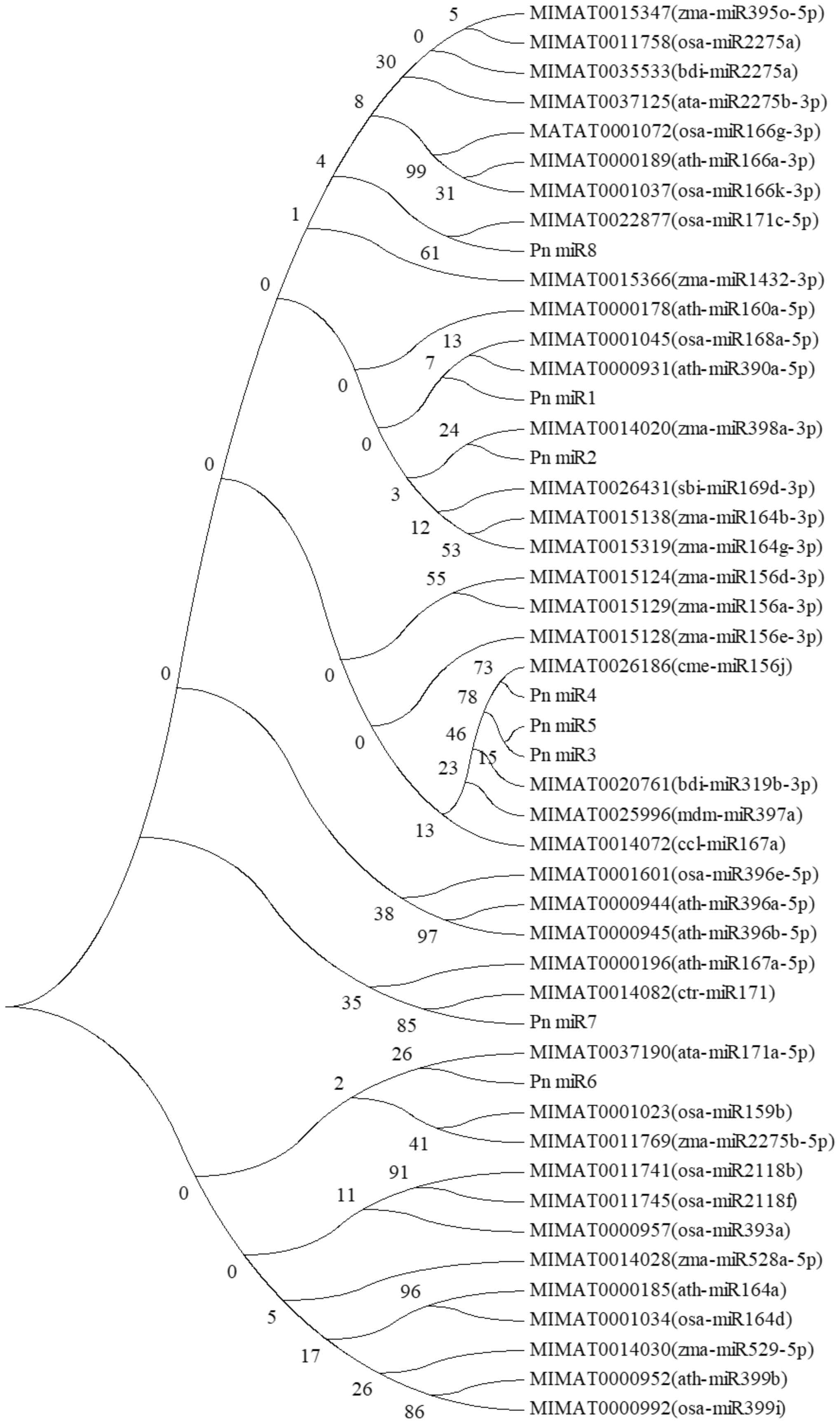

Supplement: Supplementary file 6 — Phylogenetic analysis of miRNA sequences. Phylogenetic analysis by maximum likelihood method of mature miRNA sequences expressed in the P. notatum floral transcriptome. (PDF 53 kb) [file 12864_2019_5881_MOESM6_ESM.pdf]
